# Crystal structure of bis­{4-bromo-2-[(carb­amim­id­amido­imino)­meth­yl]phenolato-κ^3^ *N*,*N*′,*O*}cobalt(III) nitrate di­methyl­formamide monosolvate

**DOI:** 10.1107/S2056989016008690

**Published:** 2016-06-10

**Authors:** Elena A. Buvaylo, Katerina A. Kasyanova, Olga Yu. Vassilyeva, Brian W. Skelton

**Affiliations:** a Department of Chemistry, Taras Shevchenko National University of Kyiv, 64/13 Volodymyrska Street, Kyiv 01601, Ukraine; b Centre for Microscopy, Characterisation and Analysis, M313, University of Western Australia, Perth, WA 6009, Australia

**Keywords:** crystal structure, monomeric octa­hedral Co^III^ complex, Schiff base ligand, amino­guanidine, 5-bromo­salicyl­aldehyde

## Abstract

The title compound, [Co(C_8_H_8_BrN_4_O)_2_]NO_3_·C_3_H_7_NO, is formed of discrete [Co*L*_2_]^+^ cations, nitrate anions and di­methyl­formamide (DMF) mol­ecules of crystallization. The cation has no crystallographically imposed symmetry. The ligand mol­ecules are deprotonated at the phenol O atom and octa­hedrally coordinate the Co^III^ atoms through the azomethine N and phenolate O atoms in a *mer* configuration. The deprotonated ligand mol­ecules adopt an almost planar conformation. In the crystal lattice, the cations are arranged in layers in the *ab* plane divided by the nitrate anions and solvent mol­ecules. No π–π stacking is observed. All of the amine H atoms are involved in hydrogen bonding to nitrate, DMF or ligand O atoms or to one of the Br atoms, forming two-dimensional networks parallel to (100).

## Abstract 2

In [Co(C_8_H_8_BrN_4_O)_2_]NO_3_·C_3_H_7_NO, the ligand mol­ecules are deprotonated at the phenol O atom and octa­hedrally coordinate the Co^III^ atoms through the azomethine N and phenolate O atoms in a *mer* configuration. In the crystal lattice, the cations are arranged in layers in the *ab* plane divided by the nitrate anions and solvent mol­ecules. All of the amine H atoms are involved in hydrogen bonding to nitrate, DMF or ligand O atoms or to one of the Br atoms, forming two-dimensional networks.

## Chemical context   

Amino­guanidine (AG) has been extensively studied as one of the most promising compounds for the treatment of diabetic complications (Thornalley, 2003 ▸). AG-based Schiff bases have attracted much research attention owing to experimental evidence that a pyridoxal-amino­guanidine Schiff base adduct exhibited advanced glycation inhibitory activity comparable to that of AG, while causing no decrease in the liver pyridoxal phosphate content of normal mice (Taguchi *et al.*, 1998 ▸, 1999 ▸). The study of the chelating properties of AG-based Schiff bases toward metal ions may help to understand the mechanism of action of drugs and possible benefits of chelation therapy in diabetes (Nagai *et al.*, 2012 ▸).

Multinuclear Schiff base metal complexes, coupled systems in particular, are also of special inter­est in materials science. During the last few years, we have been exploring the chemistry of transition metal complexes of Schiff base ligands with the aim of preparing heterometallic polynuclear compounds with diverse potential advantages. In these studies, we continued to apply *direct synthesis of coordination compounds*, an approach that employs zero-valent metal (metal oxide) as a source of metal ions along with a salt of another metal (Vinogradova *et al.*, 2001 ▸; Buvaylo *et al.*, 2009 ▸; Semenaka *et al.*, 2010 ▸; Nesterov *et al.*, 2011 ▸). The metal powder is oxidized during the synthesis by di­oxy­gen from the air. The main advantage of this approach is generation of building blocks *in situ*, in one reaction vessel, thus eliminating separate steps in building-block construction. Reactions of a metal powder and another metal salt in air with a solution containing a pre-formed Schiff base ligand yielded a number of novel Cu/Cr and Co/Fe compounds (Nikitina *et al.*, 2008 ▸; Chygorin *et al.*, 2012 ▸).
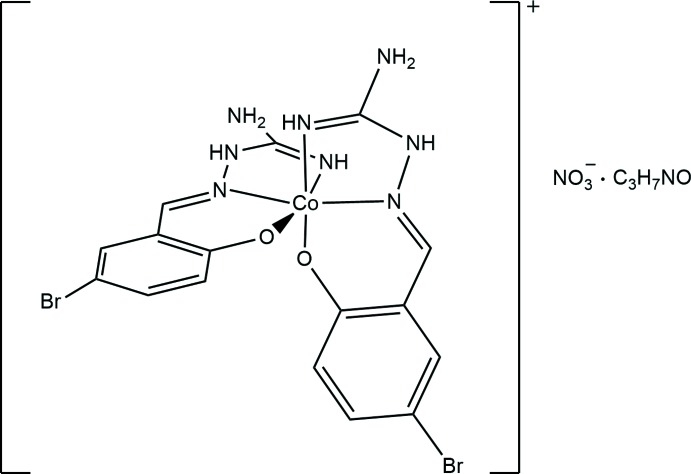


**Figure 1:**
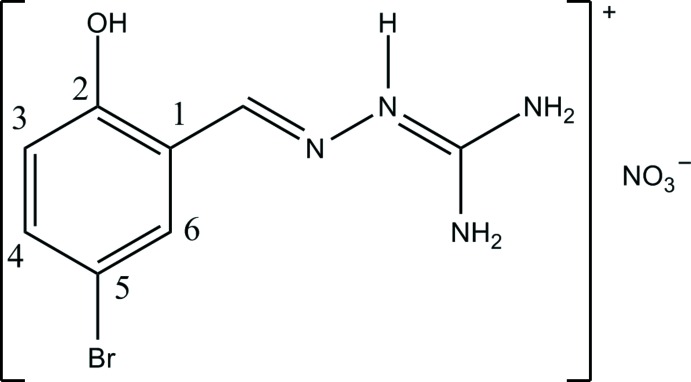
Scheme of H*L*·HNO_3_.

The title compound was isolated in an attempt to prepare a heterometallic Co/Mn compound with the ligand, H*L*·HNO_3_ (Fig. 1 ▸) that was synthesized from Schiff base formation of 5-bromo­salicyl­aldehyde with AG·HNO_3_. Mn powder and Co(NO_3_)_2_·6H_2_O were reacted with the Schiff base formed *in situ* in methanol/di­methyl­formamide (DMF) mixture in a 1:1:2 molar ratio. The isolated dark-red microcrystalline product was identified crystallographically to be the mononuclear Co^III^ Schiff base complex Co*L*_2_NO_3_·DMF (I) which did not contain any manganese.

## Structural commentary   

**Figure 2:**
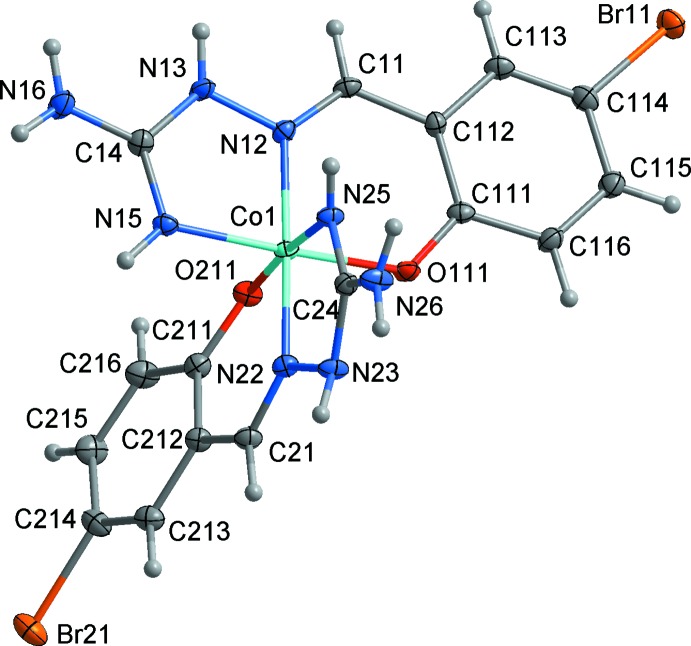
The mol­ecular structure of the title complex, showing the atom-numbering scheme. Non-H atoms are shown with displacement ellipsoids at the 50% probability level.

The title compound [Co(C_8_H_8_BrN_4_O)_2_]NO_3_·C_3_H_7_NO, (I), is formed of discrete [Co*L*_2_]^+^ cations, nitrate anions and DMF mol­ecules of crystallization. The cation has no crystallographically imposed symmetry (Fig. 2 ▸). The ligand mol­ecules are deprotonated at the phenol oxygen atom and coordinate to the Co^III^ atom through four azomethine N and two phenol O atoms in such a way that the Co^III^ atom is octa­hedrally surrounded by two anionic ligands in a *mer* configuration. The Co—N/O distances (Table 1 ▸) fall in the range 1.887 (2)–1.9135 (18) Å, the *trans* angles at the metal atom vary from 175.14 (9) to 177.14 (8)°, the *cis* angles lie in the range 82.62 (9) to 94.35 (8)°. The deprotonated ligand mol­ecules adopt an almost planar conformation.

The coordination geometry around the metal atom has a close resemblance to that found in Co^III^ complexes with a very similar ligand which results from the condensation between salicyl­aldehyde and AG hydro­chloride: bis­{2-[(guanidino­imino)­meth­yl]phenolato-κ^3^ *N*,*N*′,*O*]}cobalt(III) chloride hemihydrate (CSD refcode MEXGED; Buvaylo *et al.*, 2013 ▸), and its solvatomorph trihydrate (CSD refcode GEMJOY; Chumakov *et al.*, 2006 ▸). Co—N/O distances in MEXGED, which possesses two independent cations, vary from 1.8863 (8) to 1.9290 (8) Å, the *trans* angles at the metal atoms fall in the range 172.24 (4)–176.71 (4)°, the *cis* angles are equal to 82.33 (4)–94.86 (4)°. Obviously, the use of the 5-bromo-deriv­ative of salicyl­aldehyde in the present study does not change the coordination properties of the resulting Schiff base ligand compared to that of parent salicyl­aldehyde-amino­guanidine Schiff base.

## Supra­molecular features   

**Figure 3:**
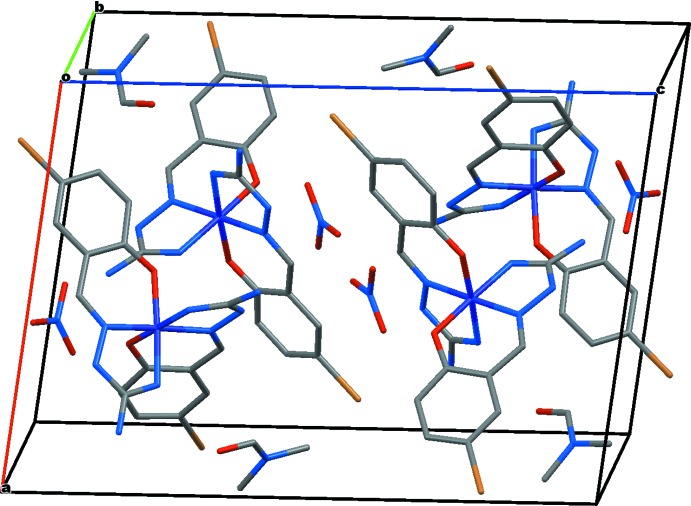
Crystal packing of (I) showing the layered arrangement of [Co*L*_2_]^+^ cations in the *ab* plane. H atoms are not shown.

**Figure 4:**
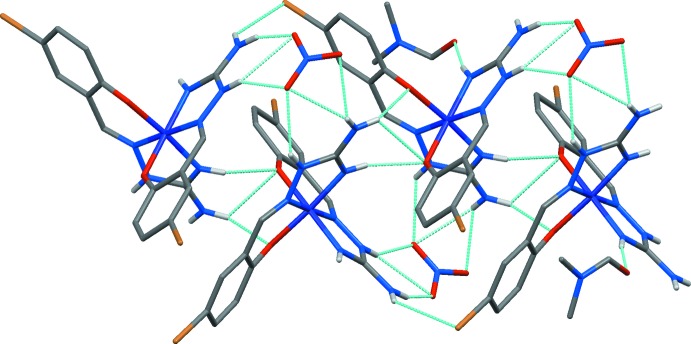
Part of the crystal structure with inter­molecular hydrogen bonds shown as blue dashed lines. CH hydrogen atoms have been omitted for clarity.

In the crystal lattice, the cations are arranged in layers in the *ab* plane divided by the nitrate anions and DMF mol­ecules (Fig. 3 ▸). Inter­actions between cations are weak, the closest Co⋯Co inter­molecular separation exceeds 5.76 Å. No π–π stacking is observed. All the amine hydrogen atoms are involved in hydrogen bonding to nitrate, DMF or ligand oxygen atoms or to one of the Br atoms, Br21, to form two-dimensional networks parallel to (100) (Fig. 4 ▸). Hydrogen-bonding geometrical details are listed in Table 2 ▸.

## Database survey   

Crystal structures of neither the ligand itself nor its metal complexes are found in the Cambridge Structure Database (Groom *et al.*, 2016 ▸; CSD Version 5.37 plus one update). Eighteen reported structures of AG-based Schiff bases deposited in the Database incorporate various chloro, fluoro, hy­droxy, meth­oxy, methyl­thio and nitro derivatives of benzaldehyde, pyridine and pyrimidine. These organic compounds exist as zwitterions as well as chloride, nitrate, acetate, di­hydrogenphosphate and sulfate salts in the solid state.

Of 18 crystal structures of Schiff base metal complexes derived from AG, six are Fe, Cu and Zn compounds that contain a pyridoxal-amino­guanidine ligand. The latter has been of much inter­est due to its suggested superiority to AG in the treatment of diabetic complications. The remaining 12 compounds are mostly mononuclear Cu^II^ complexes (four) and CuCl_4_^2–^ salts (four) with protonated Schiff base ligands as cations. Other mononuclear complexes and hybrid metal salts of AG-based Schiff base ligands comprise V, Co, and Ni, Cd structures, respectively. The Schiff base ligands derived from AG do not show any coordination variability in their metal complexes - the ligand tends to coordinate through two azomethine N atoms and phen­oxy O atom from the ring if such one is present.

## Synthesis and crystallization   

**Figure 5:**
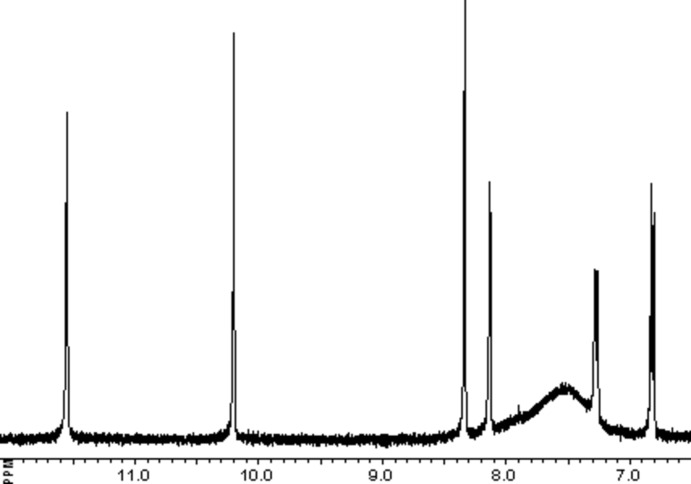
400 MHz ^1^H NMR spectrum of H*L*·HNO_3_ in DMSO-*d*_6_ at 293 K in the range 12–6.5 p.p.m.

**Synthesis of (5-bromo­salicyl­idene)amino­guanidine HNO_3_ (H** ***L***·**HNO_3_) ligand:** 5-Bromo­salicyl­aldehyde (0.40 g, 2 mmol) in ethanol (10 ml) was poured into an aqueous solution (10 ml) of AG·HNO_3_ (0.35 g, 2 mmol) and 5 drops of concentrated nitric acid were added to the resulting clear solution. It was heated to 353 K under stirring for 20 min and then cooled in air. A white crystalline precipitate of H*L*·HNO_3_ deposited shortly. It was filtered off, washed with distilled water and dried out in air (yield: 82%).^1^H NMR (400 MHz, DMSO-*d*_6_, *s*, singlet; *br*, broad; *d*, doublet; Fig. 5 ▸): 11.55, *s* (1H, phenolic OH); 10.20, *s* (1H, NH); 8.34, *s* (1H, CH=N azomethine); 8.13, *s* (1H, C-6); 7.52, *br* (4H, NH_2_); 7.27, *d* (H, C-3, *J* = 8.8 Hz); 6.82, *d* (H, C-4, *J* = 8.8 Hz). FT–IR (solid) ν (cm^−1^): 3500*w*, 3446*m*, 3418*m*, 3322*m*, 3208*s*, 3124*m*, 2922*m*, 2892*m*, 2854*m*, 2816*m*, 1692*s*, 1632*vs*, 1476*s*, 1420s, 1384vs, 1346*s*, 1336*s*, 1256*s*, 1190*m*, 1048*m*, 956*w*, 904*w*, 836*w*, 820*w*, 654*w*, 622m, 538*w*, 480*w*.

**Synthesis of 1:** Mn powder (0.03 g, 0.5 mmol), Co(NO_3_)_2_·6H_2_O (0.15 g, 0.5 mmol) and H*L*·HNO_3_ (0.32 g, 1 mmol) were added to methanol (20 ml) and the mixture was heated to 323 K under stirring until total dissolution of the manganese powder was observed (1 h). The resulting red solution was filtered and allowed to stand at room temperature. Dark-red microcrystals of the title compound were formed over several days. They were collected by filter-suction, washed with dry Pr^i^OH and finally dried *in vacuo* (yield: 39%). FT–IR (solid) ν (cm^−1^): 3476*m*, 3406*m*, 3358*m*, 3226*s*, 3180*s*, 3092*m*, 3054*m*, 2998*m*, 2940*m*, 2900*m*, 2800*m*, 1660*sh*, 1650*vs*, 1596*s*, 1556*s*, 1522*m*, 1454*s*, 1384*s*, 1354*m*, 1334*s*, 1290*s*, 1250*m*, 1182*m*, 1134*m*, 1102*m*, 1046*w*, 926*m*, 822*m*, 969*m*, 656*m*, 620*m*, 574*m*, 526*m*, 468*w*.

## Refinement   

Crystal data, data collection and structure refinement details are summarized in Table 3 ▸. All hydrogen atoms bound to carbon were included in calculated positions and refined using a riding model with isotropic displacement parameters based on those of the parent atom (C—H = 0.95 Å, *U*_iso_(H) = 1.2*U*_eq_C for CH, C—H = 0.98 Å, *U*_iso_(H) = 1.5*U*_eq_C for CH_3_). NH hydrogen atoms were refined with bond lengths restrained to ideal values (N—H = 0.88 Å). Anisotropic displacement parameters were employed for the non-hydrogen atoms.

## Supplementary Material

Crystal structure: contains datablock(s) I, global. DOI: 10.1107/S2056989016008690/hg5475sup1.cif

Structure factors: contains datablock(s) I. DOI: 10.1107/S2056989016008690/hg5475Isup2.hkl

IR spectrum of the ligand. DOI: 10.1107/S2056989016008690/hg5475sup3.tif

IR spectrum of the complex. DOI: 10.1107/S2056989016008690/hg5475sup4.tif

CCDC reference: 1482509

Additional supporting information: crystallographic information; 3D view; checkCIF report

## Figures and Tables

**Table 1:** Selected geometric parameters (Å, °)

Co1—N12	1.887 (2)	Co1—N15	1.899 (2)
Co1—N22	1.889 (2)	Co1—N25	1.902 (2)
Co1—O111	1.8919 (18)	Co1—O211	1.9135 (18)
			
N12—Co1—N22	175.76 (9)	O111—Co1—N25	88.36 (9)
N12—Co1—O111	94.35 (8)	N15—Co1—N25	92.93 (9)
N22—Co1—O111	88.42 (8)	N12—Co1—O211	89.98 (8)
N12—Co1—N15	83.02 (9)	N22—Co1—O211	93.29 (8)
N22—Co1—N15	94.27 (9)	O111—Co1—O211	88.90 (8)
O111—Co1—N15	177.14 (8)	N15—Co1—O211	89.99 (9)
N12—Co1—N25	94.23 (9)	N25—Co1—O211	175.14 (9)
N22—Co1—N25	82.62 (9)		

**Table 2:** Hydrogen-bond geometry (Å, °)

*D*—H⋯*A*	*D*—H	H⋯*A*	*D*⋯*A*	*D*—H⋯*A*
N13—H13⋯O12	0.871 (18)	1.987 (19)	2.851 (3)	171 (3)
N15—H15⋯O10	0.867 (18)	2.072 (18)	2.937 (3)	175 (3)
N16—H16*A*⋯Br21^i^	0.871 (18)	2.83 (3)	3.529 (2)	139 (3)
N16—H16*B*⋯O13	0.86 (3)	2.19 (3)	2.998 (3)	155 (4)
N23—H23⋯O11^ii^	0.878 (18)	2.00 (2)	2.854 (3)	163 (4)
N25—H25⋯O111^iii^	0.868 (17)	2.07 (2)	2.865 (3)	151 (3)
N26—H26*A*⋯O211^iii^	0.872 (17)	2.058 (19)	2.913 (3)	166 (3)
N26—H26*B*⋯O12^ii^	0.883 (19)	2.34 (3)	3.054 (3)	138 (3)

Symmetry codes: (i) 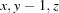; (ii) 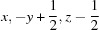; (iii) 
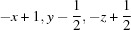
.

**Table 3:** Experimental details

Crystal data	
Chemical formula	[Co(C_8_H_8_BrN_4_O)_2_]NO_3_·C_3_H_7_NO
*M*_r_	706.22
Crystal system, space group	Monoclinic, *P*2_1_/*c*
Temperature (K)	100
*a*, *b*, *c* (Å)	13.5778 (3), 9.9492 (3), 19.0240 (4)
β (°)	98.302 (2)
*V* (Å^3^)	2542.99 (11)
*Z*	4
Radiation type	Mo *K*α
μ (mm^−1^)	3.88
Crystal size (mm)	0.23 × 0.11 × 0.11

Data collection	
Diffractometer	Oxford Diffraction Gemini
Absorption correction	Analytical [*CrysAlis PRO* (Agilent, 2014 ▸), analytical numeric absorption correction (Clark & Reid, 1995 ▸)]
*T*_min_, *T*_max_	0.771, 0.891
No. of measured, independent and observed [*I* > 2σ(*I*)] reflections	35245, 8094, 6450
*R*_int_	0.061
(sin θ/λ)_max_ (Å^−1^)	0.725

Refinement	
*R*[*F* ^2^ > 2σ(*F* ^2^)], *wR*(*F* ^2^), *S*	0.045, 0.110, 1.02
No. of reflections	8094
No. of parameters	378
No. of restraints	8
H-atom treatment	H atoms treated by a mixture of independent and constrained refinement
Δρ_max_, Δρ_min_ (e Å^−3^)	1.32, −0.68

Computer programs: *CrysAlis PRO* (Agilent, 2014 ▸), *SIR92* (Altomare *et al.*, 1994 ▸), *SHELXL2014* (Sheldrick, 2015 ▸), *Mercury* (Macrae *et al.*, 2006 ▸) and *publCIF* (Westrip, 2010 ▸).

## supplementary crystallographic information

### Crystal data

**Table:**

[Co(C_8_H_8_BrN_4_O)_2_]NO_3_·C_3_H_7_NO	*F*(000) = 1408
*M**_r_* = 706.22	*D*_x_ = 1.845 Mg m^−^^3^
Monoclinic, *P*2_1_/*c*	Mo *K*α radiation, λ = 0.71073 Å
Hall symbol: -P 2ybc	Cell parameters from 8612 reflections
*a* = 13.5778 (3) Å	θ = 2.4–32.4°
*b* = 9.9492 (3) Å	µ = 3.88 mm^−^^1^
*c* = 19.0240 (4) Å	*T* = 100 K
β = 98.302 (2)°	Prism, dark_red
*V* = 2542.99 (11) Å^3^	0.23 × 0.11 × 0.11 mm
*Z* = 4	

### Data collection

**Table:**

Oxford Diffraction Gemini diffractometer	8094 independent reflections
Radiation source: fine-focus sealed X-ray tube	6450 reflections with *I* > 2σ(*I*)
Graphite monochromator	*R*_int_ = 0.061
Detector resolution: 10.4738 pixels mm^-1^	θ_max_ = 31.0°, θ_min_ = 2.6°
ω scans	*h* = −19→19
Absorption correction: analytical [*CrysAlis PRO* (Agilent, 2014), analytical numeric absorption correction (Clark & Reid, 1995)]	*k* = −14→13
*T*_min_ = 0.771, *T*_max_ = 0.891	*l* = −27→27
35245 measured reflections	

### Refinement

**Table:**

Refinement on *F*^2^	8 restraints
Least-squares matrix: full	Hydrogen site location: mixed
*R*[*F*^2^ > 2σ(*F*^2^)] = 0.045	H atoms treated by a mixture of independent and constrained refinement
*wR*(*F*^2^) = 0.110	*w* = 1/[σ^2^(*F*_o_^2^) + (0.0549*P*)^2^ + 1.1125*P*] where *P* = (*F*_o_^2^ + 2*F*_c_^2^)/3
*S* = 1.02	(Δ/σ)_max_ = 0.002
8094 reflections	Δρ_max_ = 1.32 e Å^−^^3^
378 parameters	Δρ_min_ = −0.68 e Å^−^^3^

### Special details

**Table:**

Geometry. All esds (except the esd in the dihedral angle between two l.s. planes) are estimated using the full covariance matrix. The cell esds are taken into account individually in the estimation of esds in distances, angles and torsion angles; correlations between esds in cell parameters are only used when they are defined by crystal symmetry. An approximate (isotropic) treatment of cell esds is used for estimating esds involving l.s. planes.
Refinement. NH hydrogen atoms were refined with bond distances restrained to ideal values. Two reflections which were considered to be masked by the beam stop were omitted from the refinement. Largest peak is 0.79 Angstroms from Br21. Largest trough is 0.64 Angstroms from Co1.

### Fractional atomic coordinates and isotropic or equivalent isotropic displacement parameters (Å^2^)

**Table:**

	*x*	*y*	*z*	*U*_iso_*/*U*_eq_	
Co1	0.40679 (2)	0.49709 (3)	0.27902 (2)	0.01086 (8)	
Br11	0.84873 (2)	0.53347 (3)	0.55431 (2)	0.01994 (8)	
Br21	0.02860 (2)	1.02172 (3)	0.19557 (2)	0.02163 (8)	
C111	0.59602 (18)	0.5698 (3)	0.35767 (13)	0.0135 (5)	
O111	0.52857 (13)	0.59057 (19)	0.30171 (9)	0.0140 (4)	
C112	0.58479 (18)	0.4791 (3)	0.41364 (13)	0.0125 (5)	
C113	0.6615 (2)	0.4690 (3)	0.47318 (13)	0.0158 (5)	
H113	0.6541	0.4095	0.5112	0.019*	
C114	0.74582 (19)	0.5454 (3)	0.47532 (14)	0.0171 (5)	
C115	0.7590 (2)	0.6339 (3)	0.42019 (14)	0.0174 (5)	
H115	0.8182	0.6855	0.4224	0.021*	
C116	0.68522 (19)	0.6452 (3)	0.36274 (14)	0.0168 (5)	
H116	0.6944	0.7053	0.3254	0.02*	
C11	0.49888 (18)	0.3939 (3)	0.41404 (13)	0.0137 (5)	
H11	0.4961	0.3371	0.4538	0.016*	
N12	0.42526 (15)	0.3917 (2)	0.36236 (11)	0.0125 (4)	
N13	0.34742 (16)	0.3036 (2)	0.36593 (11)	0.0155 (4)	
C14	0.27399 (18)	0.3108 (3)	0.30883 (13)	0.0141 (5)	
N15	0.28470 (15)	0.4014 (2)	0.26114 (11)	0.0129 (4)	
N16	0.19840 (18)	0.2235 (3)	0.30737 (13)	0.0196 (5)	
C211	0.27262 (18)	0.7103 (3)	0.29499 (13)	0.0140 (5)	
O211	0.34349 (13)	0.63305 (19)	0.32785 (9)	0.0142 (4)	
C212	0.26407 (18)	0.7469 (3)	0.22191 (13)	0.0131 (5)	
C213	0.19201 (19)	0.8413 (3)	0.19325 (14)	0.0161 (5)	
H213	0.1889	0.869	0.1452	0.019*	
C214	0.1259 (2)	0.8938 (3)	0.23468 (14)	0.0183 (5)	
C215	0.1301 (2)	0.8550 (3)	0.30568 (15)	0.0199 (6)	
H215	0.0829	0.8888	0.3336	0.024*	
C216	0.2033 (2)	0.7672 (3)	0.33485 (14)	0.0190 (5)	
H216	0.2071	0.7441	0.3836	0.023*	
C21	0.32682 (18)	0.6909 (3)	0.17418 (13)	0.0144 (5)	
H21	0.3245	0.7289	0.1282	0.030 (9)*	
N22	0.38606 (15)	0.5908 (2)	0.19178 (11)	0.0120 (4)	
N23	0.44539 (17)	0.5417 (2)	0.14357 (11)	0.0142 (4)	
C24	0.48136 (18)	0.4152 (3)	0.16206 (13)	0.0132 (5)	
N25	0.47343 (16)	0.3738 (2)	0.22568 (11)	0.0128 (4)	
N26	0.52409 (17)	0.3479 (3)	0.11301 (12)	0.0172 (5)	
N1	0.33098 (17)	0.0191 (2)	0.46915 (11)	0.0156 (4)	
O11	0.34224 (14)	−0.0888 (2)	0.50414 (10)	0.0183 (4)	
O12	0.40449 (14)	0.0966 (2)	0.46727 (10)	0.0203 (4)	
O13	0.24818 (15)	0.0494 (2)	0.43623 (11)	0.0269 (5)	
C101	0.0924 (2)	0.6797 (3)	0.00598 (15)	0.0256 (6)	
H10A	0.15	0.6444	−0.0138	0.038*	
H10B	0.1041	0.774	0.0193	0.038*	
H10C	0.0328	0.6728	−0.0297	0.038*	
C102	−0.0029 (2)	0.6429 (3)	0.10642 (16)	0.0233 (6)	
H10D	0.0074	0.605	0.1545	0.035*	
H10E	−0.0663	0.6105	0.0809	0.035*	
H10F	−0.0043	0.7412	0.1093	0.035*	
N10	0.07772 (16)	0.6015 (3)	0.06882 (12)	0.0174 (5)	
C10	0.1335 (2)	0.4952 (3)	0.08995 (15)	0.0207 (6)	
H10	0.1856	0.4733	0.0635	0.025*	
O10	0.12327 (15)	0.4225 (2)	0.14103 (10)	0.0229 (4)	
H23	0.426 (3)	0.562 (4)	0.0989 (11)	0.043 (11)*	
H25	0.488 (2)	0.2892 (19)	0.2306 (16)	0.011 (7)*	
H26A	0.556 (2)	0.275 (2)	0.1271 (15)	0.015 (8)*	
H26B	0.522 (3)	0.378 (4)	0.0691 (12)	0.050 (12)*	
H13	0.358 (3)	0.239 (3)	0.3968 (17)	0.038 (11)*	
H15	0.2362 (18)	0.412 (4)	0.2266 (13)	0.023 (9)*	
H16A	0.1505 (19)	0.219 (4)	0.2716 (14)	0.027 (9)*	
H16B	0.200 (3)	0.157 (4)	0.337 (2)	0.067 (15)*	

### Atomic displacement parameters (Å^2^)

**Table:**

	*U*^11^	*U*^22^	*U*^33^	*U*^12^	*U*^13^	*U*^23^
Co1	0.01350 (17)	0.01171 (17)	0.00748 (15)	−0.00003 (12)	0.00193 (12)	0.00055 (11)
Br11	0.01947 (14)	0.02185 (16)	0.01635 (14)	−0.00175 (10)	−0.00467 (10)	−0.00023 (10)
Br21	0.02097 (15)	0.02263 (16)	0.01907 (14)	0.00852 (10)	−0.00461 (10)	−0.00454 (10)
C111	0.0173 (12)	0.0106 (12)	0.0125 (11)	0.0010 (9)	0.0022 (9)	−0.0001 (9)
O111	0.0175 (9)	0.0141 (10)	0.0097 (8)	−0.0021 (7)	−0.0001 (6)	0.0029 (7)
C112	0.0105 (11)	0.0154 (13)	0.0114 (11)	−0.0003 (9)	0.0012 (8)	−0.0009 (9)
C113	0.0205 (13)	0.0170 (14)	0.0097 (11)	0.0005 (10)	0.0019 (9)	0.0012 (9)
C114	0.0173 (12)	0.0190 (14)	0.0142 (12)	0.0016 (10)	−0.0007 (10)	−0.0005 (10)
C115	0.0173 (12)	0.0167 (14)	0.0182 (12)	−0.0029 (10)	0.0025 (10)	−0.0001 (10)
C116	0.0179 (12)	0.0171 (14)	0.0157 (12)	−0.0037 (10)	0.0036 (10)	0.0027 (10)
C11	0.0185 (12)	0.0127 (13)	0.0101 (11)	−0.0004 (9)	0.0029 (9)	0.0018 (9)
N12	0.0141 (10)	0.0125 (11)	0.0113 (9)	−0.0016 (8)	0.0030 (8)	−0.0004 (8)
N13	0.0175 (11)	0.0168 (12)	0.0115 (10)	−0.0050 (8)	−0.0002 (8)	0.0050 (8)
C14	0.0153 (12)	0.0166 (13)	0.0107 (11)	−0.0008 (9)	0.0026 (9)	−0.0015 (9)
N15	0.0147 (10)	0.0139 (11)	0.0096 (9)	0.0003 (8)	0.0001 (8)	0.0010 (8)
N16	0.0191 (12)	0.0234 (14)	0.0155 (11)	−0.0078 (9)	0.0000 (9)	0.0024 (9)
C211	0.0164 (12)	0.0139 (13)	0.0118 (11)	−0.0010 (9)	0.0024 (9)	−0.0015 (9)
O211	0.0181 (9)	0.0147 (10)	0.0099 (8)	0.0024 (7)	0.0029 (7)	−0.0017 (7)
C212	0.0151 (11)	0.0126 (12)	0.0115 (11)	0.0009 (9)	0.0021 (9)	−0.0007 (9)
C213	0.0207 (13)	0.0142 (13)	0.0127 (11)	0.0000 (10)	0.0001 (9)	−0.0012 (9)
C214	0.0185 (13)	0.0160 (14)	0.0185 (13)	0.0034 (10)	−0.0036 (10)	−0.0025 (10)
C215	0.0230 (14)	0.0199 (15)	0.0185 (13)	0.0050 (10)	0.0088 (11)	−0.0011 (10)
C216	0.0246 (14)	0.0207 (15)	0.0128 (12)	0.0052 (11)	0.0062 (10)	−0.0001 (10)
C21	0.0173 (12)	0.0155 (13)	0.0108 (11)	0.0006 (9)	0.0028 (9)	0.0014 (9)
N22	0.0154 (10)	0.0119 (11)	0.0091 (9)	−0.0003 (8)	0.0027 (7)	−0.0008 (7)
N23	0.0196 (11)	0.0149 (11)	0.0088 (9)	0.0041 (8)	0.0050 (8)	0.0019 (8)
C24	0.0129 (11)	0.0145 (13)	0.0123 (11)	−0.0014 (9)	0.0017 (9)	0.0011 (9)
N25	0.0161 (10)	0.0130 (11)	0.0098 (9)	0.0014 (8)	0.0037 (8)	0.0015 (8)
N26	0.0229 (12)	0.0190 (12)	0.0115 (10)	0.0070 (9)	0.0083 (9)	0.0031 (9)
N1	0.0179 (11)	0.0207 (12)	0.0089 (10)	−0.0002 (8)	0.0043 (8)	−0.0001 (8)
O11	0.0232 (10)	0.0198 (11)	0.0125 (9)	−0.0019 (8)	0.0043 (7)	0.0039 (7)
O12	0.0192 (10)	0.0215 (11)	0.0202 (10)	−0.0032 (8)	0.0024 (7)	0.0033 (8)
O13	0.0178 (10)	0.0392 (14)	0.0226 (11)	−0.0004 (9)	−0.0014 (8)	0.0117 (9)
C101	0.0290 (16)	0.0304 (18)	0.0189 (14)	−0.0005 (12)	0.0082 (12)	0.0085 (12)
C102	0.0231 (14)	0.0242 (16)	0.0245 (14)	0.0002 (11)	0.0096 (11)	−0.0010 (12)
N10	0.0183 (11)	0.0191 (13)	0.0156 (10)	0.0013 (9)	0.0052 (8)	0.0021 (8)
C10	0.0178 (13)	0.0262 (16)	0.0182 (13)	0.0007 (11)	0.0031 (10)	−0.0007 (11)
O10	0.0239 (10)	0.0259 (12)	0.0188 (10)	0.0003 (8)	0.0022 (8)	0.0053 (8)

### Geometric parameters (Å, º)

**Table:**

Co1—N12	1.887 (2)	C212—C213	1.408 (4)
Co1—N22	1.889 (2)	C212—C21	1.444 (3)
Co1—O111	1.8919 (18)	C213—C214	1.380 (4)
Co1—N15	1.899 (2)	C213—H213	0.95
Co1—N25	1.902 (2)	C214—C215	1.398 (4)
Co1—O211	1.9135 (18)	C215—C216	1.379 (4)
Br11—C114	1.902 (3)	C215—H215	0.95
Br21—C214	1.906 (3)	C216—H216	0.95
C111—O111	1.317 (3)	C21—N22	1.294 (3)
C111—C116	1.416 (4)	C21—H21	0.95
C111—C112	1.420 (4)	N22—N23	1.393 (3)
C112—C113	1.427 (4)	N23—C24	1.377 (3)
C112—C11	1.443 (3)	N23—H23	0.878 (18)
C113—C114	1.371 (4)	C24—N25	1.298 (3)
C113—H113	0.95	C24—N26	1.346 (3)
C114—C115	1.400 (4)	N25—H25	0.868 (17)
C115—C116	1.376 (4)	N26—H26A	0.872 (17)
C115—H115	0.95	N26—H26B	0.883 (19)
C116—H116	0.95	N1—O13	1.242 (3)
C11—N12	1.297 (3)	N1—O11	1.261 (3)
C11—H11	0.95	N1—O12	1.266 (3)
N12—N13	1.382 (3)	C101—N10	1.464 (3)
N13—C14	1.366 (3)	C101—H10A	0.98
N13—H13	0.871 (18)	C101—H10B	0.98
C14—N15	1.302 (3)	C101—H10C	0.98
C14—N16	1.342 (3)	C102—N10	1.452 (3)
N15—H15	0.867 (18)	C102—H10D	0.98
N16—H16A	0.871 (18)	C102—H10E	0.98
N16—H16B	0.86 (3)	C102—H10F	0.98
C211—O211	1.317 (3)	N10—C10	1.328 (4)
C211—C216	1.410 (3)	C10—O10	1.235 (3)
C211—C212	1.426 (3)	C10—H10	0.95
			
N12—Co1—N22	175.76 (9)	C211—O211—Co1	122.07 (15)
N12—Co1—O111	94.35 (8)	C213—C212—C211	120.1 (2)
N22—Co1—O111	88.42 (8)	C213—C212—C21	117.0 (2)
N12—Co1—N15	83.02 (9)	C211—C212—C21	122.8 (2)
N22—Co1—N15	94.27 (9)	C214—C213—C212	120.3 (2)
O111—Co1—N15	177.14 (8)	C214—C213—H213	119.9
N12—Co1—N25	94.23 (9)	C212—C213—H213	119.9
N22—Co1—N25	82.62 (9)	C213—C214—C215	120.5 (2)
O111—Co1—N25	88.36 (9)	C213—C214—Br21	120.1 (2)
N15—Co1—N25	92.93 (9)	C215—C214—Br21	119.4 (2)
N12—Co1—O211	89.98 (8)	C216—C215—C214	119.4 (2)
N22—Co1—O211	93.29 (8)	C216—C215—H215	120.3
O111—Co1—O211	88.90 (8)	C214—C215—H215	120.3
N15—Co1—O211	89.99 (9)	C215—C216—C211	122.4 (2)
N25—Co1—O211	175.14 (9)	C215—C216—H216	118.8
O111—C111—C116	117.4 (2)	C211—C216—H216	118.8
O111—C111—C112	124.7 (2)	N22—C21—C212	122.4 (2)
C116—C111—C112	118.0 (2)	N22—C21—H21	118.8
C111—O111—Co1	126.17 (16)	C212—C21—H21	118.8
C111—C112—C113	119.7 (2)	C21—N22—N23	119.8 (2)
C111—C112—C11	123.5 (2)	C21—N22—Co1	127.99 (17)
C113—C112—C11	116.9 (2)	N23—N22—Co1	112.24 (16)
C114—C113—C112	119.7 (2)	C24—N23—N22	111.7 (2)
C114—C113—H113	120.2	C24—N23—H23	120 (3)
C112—C113—H113	120.2	N22—N23—H23	116 (3)
C113—C114—C115	121.5 (2)	N25—C24—N26	126.2 (2)
C113—C114—Br11	120.3 (2)	N25—C24—N23	117.0 (2)
C115—C114—Br11	118.2 (2)	N26—C24—N23	116.8 (2)
C116—C115—C114	119.3 (2)	C24—N25—Co1	113.73 (18)
C116—C115—H115	120.3	C24—N25—H25	111 (2)
C114—C115—H115	120.3	Co1—N25—H25	133.7 (19)
C115—C116—C111	121.8 (2)	C24—N26—H26A	117 (2)
C115—C116—H116	119.1	C24—N26—H26B	122 (3)
C111—C116—H116	119.1	H26A—N26—H26B	121 (3)
N12—C11—C112	122.8 (2)	O13—N1—O11	120.3 (2)
N12—C11—H11	118.6	O13—N1—O12	119.9 (2)
C112—C11—H11	118.6	O11—N1—O12	119.8 (2)
C11—N12—N13	119.0 (2)	N10—C101—H10A	109.5
C11—N12—Co1	128.42 (18)	N10—C101—H10B	109.5
N13—N12—Co1	112.57 (16)	H10A—C101—H10B	109.5
C14—N13—N12	113.8 (2)	N10—C101—H10C	109.5
C14—N13—H13	127 (3)	H10A—C101—H10C	109.5
N12—N13—H13	117 (2)	H10B—C101—H10C	109.5
N15—C14—N16	126.6 (2)	N10—C102—H10D	109.5
N15—C14—N13	116.6 (2)	N10—C102—H10E	109.5
N16—C14—N13	116.8 (2)	H10D—C102—H10E	109.5
C14—N15—Co1	113.87 (17)	N10—C102—H10F	109.5
C14—N15—H15	118 (2)	H10D—C102—H10F	109.5
Co1—N15—H15	128 (2)	H10E—C102—H10F	109.5
C14—N16—H16A	122 (2)	C10—N10—C102	121.0 (2)
C14—N16—H16B	122 (3)	C10—N10—C101	122.1 (2)
H16A—N16—H16B	114 (4)	C102—N10—C101	116.9 (2)
O211—C211—C216	118.5 (2)	O10—C10—N10	125.5 (3)
O211—C211—C212	124.3 (2)	O10—C10—H10	117.3
C216—C211—C212	117.1 (2)	N10—C10—H10	117.3

### Hydrogen-bond geometry (Å, º)

**Table:**

*D*—H···*A*	*D*—H	H···*A*	*D*···*A*	*D*—H···*A*
N13—H13···O12	0.871 (18)	1.987 (19)	2.851 (3)	171 (3)
N15—H15···O10	0.867 (18)	2.072 (18)	2.937 (3)	175 (3)
N16—H16*A*···Br21^i^	0.871 (18)	2.83 (3)	3.529 (2)	139 (3)
N16—H16*B*···O13	0.86 (3)	2.19 (3)	2.998 (3)	155 (4)
N23—H23···O11^ii^	0.878 (18)	2.00 (2)	2.854 (3)	163 (4)
N25—H25···O111^iii^	0.868 (17)	2.07 (2)	2.865 (3)	151 (3)
N26—H26*A*···O211^iii^	0.872 (17)	2.058 (19)	2.913 (3)	166 (3)
N26—H26*B*···O12^ii^	0.883 (19)	2.34 (3)	3.054 (3)	138 (3)

Symmetry codes: (i) *x*, *y*−1, *z*; (ii) *x*, −*y*+1/2, *z*−1/2; (iii) −*x*+1, *y*−1/2, −*z*+1/2.
